# Phylogenetic analysis of Croatian orf viruses isolated from sheep and goats

**DOI:** 10.1186/1743-422X-7-314

**Published:** 2010-11-12

**Authors:** Ivana Lojkic, Zeljko Cac, Ana Beck, Tomislav Bedekovic, Zeljko Cvetnic, Branko Sostaric

**Affiliations:** 1 Department of Virology, Croatian Veterinary Institute, Savska cesta 143, 10000 Zagreb, Croatia; 2 Department of General Pathology and Pathological Morphology, Faculty of Veterinary Medicine, University of Zagreb, Heinzelova 55, 10000 Zagreb, Croatia; 3 Department for Bacteriology and Parasitology, Croatian Veterinary Institute, Savska cesta 143, 10000 Zagreb, Croatia; 4 Department for Pathology, Croatian Veterinary Institute, Savska cesta 143, 10000 Zagreb, Croatia

## Abstract

**Background:**

The *Orf virus *(ORFV) is the prototype of the parapoxvirus genus and it primarily causes contagious ecthyma in goats, sheep, and other ruminants worldwide. In this paper, we described the sequence and phylogenetic analysis of the B2L gene of ORFV from two natural outbreaks: i) in autochthonous Croatian Cres-breed sheep and ii) on small family goat farm.

**Results:**

Sequence and phylogenetic analyses of the ORFV B2L gene showed that the Cro-Cres-12446/09 and Cro-Goat-11727/10 were not clustered together. Cro-Cres-12446/09 shared the highest similarity with ORFV NZ2 from New Zealand, and Ena from Japan; Cro-Goat-11727/10 was closest to the HuB from China and Taiping and Hoping from Taiwan.

**Conclusion:**

Distinct ORFV strains are circulating in Croatia. Although ORFV infections are found ubiquitously wherever sheep and goats are farmed in Croatia, this is the first information on genetic relatedness of any Croatian ORFV with other isolates around the world.

## Background

Genus *Parapoxvirus *(PPV) has four members, *Orf virus *(ORFV), *bovine popular stomatitis virus *(BPSV), *pseudocowpox virus *(PCPV), *and parapoxvirus of red deer in New Zealand *(PVNZ). The ORFV is the prototype of the *Parapoxvirus *Genus and it primarily causes contagious ecthyma in goats, sheep, and other ruminants worldwide. Spread of infection can be by direct contact or through exposure to contaminated feeding troughs and similar fomites, including wheat stubble and thorny plants. The viruses are sometimes transmissible to humans due to direct contact [1].

Contagious ecthyma, also known as Orf, contagious pustular dermatitis, infectious labial dermatitis, scabby mouth, or sore mouth, is present in any part of the world where sheep and goats are raised [2].

The ORFV is an epitheliotropic virus that generally causes proliferative and self-limiting lesions in the skin of the lips, around the nostrils, oral mucosa and sometimes also affects the gums and tongue, especially in young lambs. Lesions can also be found occasionally on the teats of nursing animals and rarely on other organs [3]. Depending on the location of the lesions, animals may be unwilling to nurse, eat, or walk. Primary lesions usually resolve spontaneously within 3-4 weeks [4].

The disease has an economic impact on sheep farmers due to decreases in production and also has a considerable negative effect on animal welfare. In spite of the high morbidity mortality rates of up to 10% and 93% have been reported in lambs and kids, respectively [5,6].

Parapoxviruses are antigenically and genetically related and have a similar morphology, genomic organization and virulence mechanism [7]. Parapoxviruses are morphologically distinguished from other poxviruses by their ovoid shape, the crisscross pattern on the particle surface, relatively small size and high G+C content of the genome [8,9]. The ORFV genome consists of linear double-stranded DNA, it is 138 kbp and contains 132 putative genes [9]. The envelope gene (B2L) of the ORFV encodes for a highly immunogenic major envelope protein of about 42 kDa, which is a homologue of vaccinia virus major envelope antigen p37K [10]. The B2L gene has been used for the detection, molecular characterization and phylogenetic analysis of ORFV [11-13].

ORFV infections are found ubiquitously wherever sheep and goats are farmed in Croatia, since 1949, when it was recorded first time [14]. Although outbreaks of Orf have occurred, there were no reports available of the molecular diagnosis, characterization and phylogenetic analysis of the viruses involved.

In this paper, we described the sequence and phylogenetic analysis of the B2L gene of ORFVs from two natural outbreaks of infection: i) in autochthonous Croatian Cres-breed sheep and ii) on small family goat farm. This is the first information on genetic relatedness of any Croatian ORFV with other isolates around the world.

## Results

Affected animals from a Cres-breed free-range sheep flock presented typical ORFV mucosal and skin lesions. Ulcerations were presented at the nasal and oral mucosa accompanied with pustular dermatitis and severe yellowish to brownish crust formations on the lip commissures with spread to the muzzle and nostrils, ear tip skin and occasionally trunk skin. Morbidity among the lambs (3 weeks up to 2 months old) and ewes was 100% and 40%, respectively. Mortality due to starvation was recorded in 80% of affected lambs owing to suckling difficulty. Affected animals from a small family goat farm were suffering from typical ORFV ulcerations at the oral and nasal mucosa, and crust formations on doe teats. Morbidity in does and kids were 50% and 100%, respectively; recovery from disease took 3-4 weeks. No human infections were reported during both outbreaks.

A polymerase chain reaction (PCR) method using primers to amplify part of the ORFV B2L gene was used to amplify a specific fragment (594 bp) from field specimens of affected animals. Sequence analyses of the nucleotide and deduced amino acid of the ORFV B2L gene showed that the Cro-Cres-12446/09 (HQ215589) shared close relationship with other ORFV isolates from different regions (96,5-99.5% and 96,9-100%) and shared the highest homology with NZ2 from New Zealand, and Ena from Japan (99.5% and 100% at the nucleotide and aminoacid level, respectively). Cro-Goat-11727/10 (HQ215588) shared the highest similarity with NE1 isolate from Brazil, HuB from China and Taiping and Hoping goat isolates from Taiwan. The percent identities and diversities of the nucleotide sequence of the B2L gene among the different strains of ORFV were shown in Table 1. Our viruses differ from each other in 8 nucleotides and only two amino acids, on analysed fragment. The deduced amino acid sequences did not revealed any unique substitution.

**Table 1:** The percentages of identities and diversities of nucleotide sequences of the B2L gene among analysed ORFV strains.

**Virus accession no**.	**1**	**2**	**3**	**4**	**5**	**6**	**7**	**8**	**9**	**10**	**11**	**12**	**13**	**14**	**15**	**16**	**17**	**18**	**19**	**20**	
FJ808074		97,9	97,5	97,7	97,7	98,9	96,7	97,5	94,7	83,1	97,5	97,7	97,7	97,5	97,3	97,3	97,3	97,7	95,5	97,9	1
FJ665819	2,0		98,7	99,3	99,7	98,3	97,1	97,7	94,9	84,3	98,7	98,5	98,9	98,7	99,3	99,3	98,5	98,9	95,7	99,1	2
FJ665818	2,4	1,2		98,9	98,9	97,9	96,9	97,7	94,1	83,7	99,5	99,3	99,7	99,5	98,5	98,5	99,3	99,7	94,9	98,7	3
EU935106	2,2	0,6	1,0		99,1	98,1	96,9	97,5	94,3	83,9	98,9	98,7	99,1	98,9	99,1	99,1	98,7	99,1	95,1	99,3	4
EU327506	2,2	0,2	1,0	0,8		98,1	96,9	97,9	94,7	84,3	98,9	98,7	99,1	98,9	99,1	99,1	98,7	99,1	95,5	98,9	5
DQ263305	1,0	1,6	2,0	1,8	1,8		97,1	98,1	95,3	83,5	97,9	98,1	98,1	97,9	97,7	97,7	97,7	98,1	96,1	98,3	6
DQ263303	3,3	2,9	3,1	3,1	3,1	2,9		96,1	93,7	82,1	96,7	96,9	96,9	96,7	96,9	97,1	96,5	96,9	94,5	97,1	7
AY958203	2,4	2,2	2,2	2,4	2,0	1,8	3,9		94,7	83,1	97,9	98,1	97,9	97,7	97,1	97,1	97,5	97,9	95,5	97,7	8
AY453656	5,4	5,2	6,1	5,8	5,4	4,8	6,5	5,4		83,1	94,1	94,3	94,3	94,1	94,3	94,3	93,9	94,3	98,7	94,5	9
AY424973	19,1	17,6	18,3	18,1	17,6	18,6	20,4	19,1	19,1		83,9	83,7	83,9	83,7	83,7	83,7	83,7	83,9	83,7	83,7	10
AY386263	2,4	1,2	0,4	1,0	1,0	2,0	3,3	2,0	6,1	18,1		99,7	99,7	99,5	98,5	98,5	99,3	99,7	94,9	98,7	11
AY278209	2,2	1,4	0,6	1,2	1,2	1,8	3,1	1,8	5,8	18,3	0,2		99,5	99,3	98,3	98,3	99,1	99,5	95,1	98,5	12
AB521175	2,2	1,0	0,2	0,8	0,8	1,8	3,1	2,0	5,8	18,1	0,2	0,4		99,7	98,7	98,7	99,5	100	95,1	98,9	13
AB189670	2,4	1,2	0,4	1,0	1,0	2,0	3,3	2,2	6,1	18,3	0,4	0,6	0,2		98,5	98,5	99,3	99,7	94,9	98,7	14
GU320351	2,7	0,6	1,4	0,8	0,8	2,2	3,1	2,9	5,8	18,3	1,4	1,6	1,2	1,4		99,1	98,3	98,7	95,1	98,9	15
HQ215588	2,7	0,6	1,4	0,8	0,8	2,2	2,9	2,9	5,8	18,3	1,4	1,6	1,2	1,4	0,8		98,3	98,7	95,1	98,9	16
HQ215589	2,7	1,4	0,6	1,2	1,2	2,2	3,5	2,4	6,3	18,3	0,6	0,8	0,4	0,6	1,6	1,6		99,5	94,7	98,5	17
AY453667	2,2	1,0	0,2	0,8	0,8	1,8	3,1	2,0	5,8	18,1	0,2	0,4	0,0	0,2	1,2	1,2	0,4		95,1	98,9	18
AY424972	4,6	4,3	5,2	5,0	4,6	3,9	2,6	4,6	1,2	18,3	5,2	5,0	5,0	5,2	5,0	5,0	5,4	5,0		95,3	19
AY453654	2,0	0,8	1,2	0,6	1,0	1,6	2,9	2,2	5,6	18,3	1,2	1,4	1,0	1,2	1,0	1,0	1,5	1,0	4,8		20

**Figure 1:**
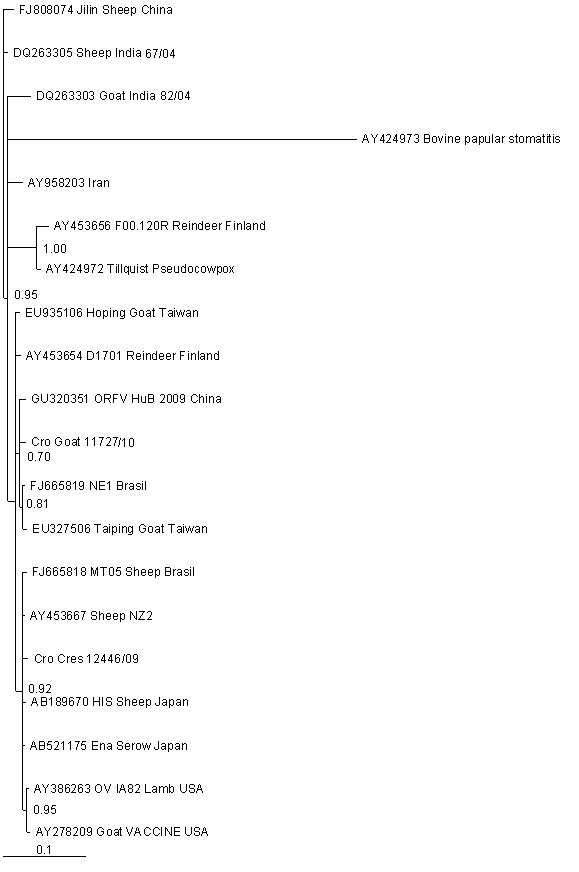
**Phylogenetic analysis of different parapoxviruses based on the partial nucleotide sequence of ORFV B2L gene**. The phylogenetic relationship was calculated using Bayesian Inference analysis. The Cro-Cres-12446/09 and Cro-Goat-11727/10 has accession numbers HQ215589, and HQ215588, respectively. Posterior probability values are shown for all nodes.

Phylogenetic analysis of parapox virus nucleotide sequences produced the similar branching patterns by both different methods: the Neighbor-joining (NJ) and the Bayesian inference (Figure 1). Posterior probabilities of MrBayes tree (Figure 1) showed good support for the three main parapox lineages: ORFV, PCPV and BPSV. Croatian viruses clustered only with ORFV. The strains of Orf lineage formed two sub-clusters; Croatian isolate Cro-Cres-12446/09 were clustered together with viruses from New Zealand, Japan and Brazil (AY453667, AB189670, AB521175, FJ665818) and Cro-Goat-11727/10 with HuB isolate from China (GU320351), another Brazilian isolate NE1 (FJ665819) and Taiwanese isolate Taiping (EU327506) (Figure 1).

## Discussion

The ORFV is common in Croatia, but is not reported in the literature because of its low morbidity and minimal economic consequences. Accordingly, immunisation of sheep and goat is not practised. It is usually diagnosed based on pathologic examinations and clinical signs. Although Croatia has a number of sheep and goat farms, Croatian islands and coast are the most representative breeding regions, with 56% from the total number of registered ewes and rams [15]. Cres-breed sheep with multiple production traits (meat-milk) is characteristic for Croatian islands Cres and Lošinj where almost 60% of territory is covered with rocky pastures, karst and poor vegetation.

In our studies we analyzed the nucleotide sequences of a fragment within the B2L gene of ORFV from two natural outbreaks of the Orf, one among sheep in the island of Cres, and other one in goats from continental part of country. The studies on genetic diversity of ORFV existing in the sheep and/or goat population in Croatia were not carried out before. Recent studies on genetic diversity of ORFV strains and isolates from small ruminants were carried out in China [16,17], India [11,18], Brazil [19] and Egypt [20].

Phylogenetic analysis of parapox virus nucleotide sequences produced the similar branching patterns by both different methods: the Neighbor-joining and the Bayesian inference. However MrBayes tree was the most accurate tree out of two calculated trees, so we chose it as a reference tree in study (Figure 1). The grouping pattern in which all the parapoxviruses - ORFV, PCPV and BPSV - formed separate clusters was consistent with report of Tikkanen et al. [13]. In the phylogenetic tree, the viruses under study grouped only with ORFV.

Our study demonstrated that Cro-Cres-12446/09 and Cro-Goat-11727/10 are not in same cluster. Cro-Cres-12446/09 is grouped with isolates from New Zealand, Brazil and Japan. Vaccinal and OV IA82 forms a subgroup within this sub-cluster, which is well supported in Bayesian tree (95%), and by the divergence factor (average 1,4) from other viruses in this cluster. Cro-Goat-11727/10 is grouped with isolates from China, Taiwan and Brazil. The first group is sharing two aminoacid sites differ from the second group. Protein sequences variations between ORFV isolates are already detected [9]. These amino acid substitutions among two groups could also be attributed to their separate geographical or evolutionary origin. Since Cres and continental viruses are not in same sub-cluster, the bigger number of available sequences in the GenBank from the neighbouring countries would be of help in explanation of the genetic relatedness of Orf viruses. For that reason it is hard to explain the origin of ORFV on a goat farm. That farm is the only one in that area, and farmers are regularly buying hay from the neighbouring regions. According to the veterinarian in charge, no contagious echtyma was recorded in that region in last 10 years. Concerning to the origin of ORFV in Cres-breed sheep, it is interesting that in 1995, due to devastation of sheep husbandry during the war, Australia and New Zealand contributed sheep to Croatia. Sheep were transported in Croatian region Lika. Unfortunately, we didn't find the valuable data if some of the sheep were actually brought to this island, which is likely because Lika is only 100 kilometres away and sheep farmers from Cres are regularly buying hay in Lika. It is possible that some of the imported sheep were persistently infected when introduced into new herd. Also, the long-lasting and stressed transportation of sheep from Australia could cause disease afterwards, and release of the virus to environment. It is well documented that viruses from *Poxviridae *family show a high environmental stability and stay contagious over a period of several months in an ambient environment. Their high resistance to drying is even enhanced by materials in which they are released into the environment (e.g., dermal crusts, serum, blood residues and other excretions [21,22].

We must emphasize that the Cres-breed sheep flock (which is normally held free-ranged) were held in small enclosure before the lambing onset, due to protection from wild boars attacks on lambs. Agglomeration of the entire flock in restricted area leaded to poor husbandry and close contact between persistently infected ewes and lambs which provided pronounced spread of virus. Persistently infected animals showing no clinical disease have been described [23] and it is possible that such animals contribute to the inter-epidemic survival of the virus. Regarding to the sequence similarity with viruses from New Zealand, Brazil and Japan, we could presume the origin of our virus from that part of the world, but not the exact way of its transmission. For the origin of Cro-Goat-11727/10 we can also suggest one of two described scenarios: contamination of hay or persistent infection. As we mentioned before, the bigger number of available sequences in the GenBank from the Croatia's neighbouring countries would be of help in explanation of the evolutionary or geographical origin and genetic relatedness of our ORFVs.

## Conclusion

In the present report, we described a severe outbreaks of contagious ecthyma in Cres-breed sheep flock and on small family goat farm and identify the causative agent as an ORFV that is genetically closely related with other ORFV isolates from distant geographical regions. Although Orf is endemic in Croatia, before this report there was no information on genetic relatedness of any Croatian ORFV with other isolates around the world.

## Methods

Tissue samples were taken from four lambs found dead originated from a farm of Cres-breed free-range sheep from Island of Cres (coordinates: 44°51' 52" North, 14°23' 67" East). The flock sized 400 ewes and 300 lambs suffering from contagious ecthyma in lambing season from January to March 2009.

Another material was taken in March 2010 from a doe originated from a small family goat farm in continental part of Croatia (Turopolje region) (coordinates: 45°39' 32" North, 15°57' 48" East). The herd sized 16 does and 4 kids suffering from typical ORFV ulcerations at the oral and nasal mucosa, and crust formations on doe teats.

Scabs, formed over the lesions (approximately 3-5 scabs per animal) were collected and 10% suspension in phosphate-buffered saline (PBS), pH 7.2, was prepared. DNA was extracted from suspension using the NucleoSpin Tissue Kit (Machery-nagel, Duren, Germany) according to the manufacturer's instructions. A set of primers: PPP-1 (5'-gtc gtc cac gat gag gag ct-3) and PPP-4 (5'-tac gtg gga agc gcc tcg ct-3), which amplify the 594 bp fragment was used in this study. These primers were designed by Inoshima et al. [12] based on the previously published sequence of the B2L gene of ORFV isolate NZ2.

The PCR reaction (total volume of 50 μl) contained 100 ng of extracted DNA, 25 μl JumpStartTM REDTaq^®^ReadyMix TM (Sigma-Aldrich, Steinheim, Germany) and 0.20 μM of each primer. Thermal cycling parameters were: initial denaturation at 94°C for 2 min, then 35 cycles of: denaturation at 94°C for 35 sec, annealing at 60°C for 35 sec, and extension at 72 °C for 45 sec, followed by the final extension at 72°C for 5 min. The reaction products were analyzed by 1,5% agarose gel electrophoresis and stained with ethidium-bromide. The PCR products that showed expected amplicon length were considered positive and purified by Exosap (USB, Staufen, Germany). Sequencing was performed in both directions by Macrogen Inc. (Seoul, Korea). The partial sequence of the major envelope gene of our viruses Cro-Cres-12446/09 and Cro-Goat-11727/10 were submitted to GenBank with accession numbers HQ215589, and HQ215588, respectively and compared with 18 different parapoxvirus strains including *Bovine papular stomatitis virus *(BPSV) and *Pseudocowpox virus *(PCPV). Detailed information of analysed sequences was shown in Table 2.

**Table 2:** Detailed information about the ORFV used in the study.

Virus	Species affected	Accession number	Country of isolation
Jilin	Sheep	FJ808074	China
NE1	Goat	FJ665819	Brazil
MT05	Sheep	FJ665818	Brazil
Hoping	Goat	EU935106	Taiwan
Taiping	Goat	EU327506	Taiwan
67/04	Sheep	DQ263305	India
82/04	Goat	DQ263303	India
-	-	AY958203	Iran
F00.120R	Reindeer	AY453656	Finland
Bovine papular stomatitis	Calf	AY424973	-
OV-IA82	Lamb	AY386263	USA
Vaccine strain	Goat	AY278209	USA
Ena	Serow	AB521175	Japan
HIS	Sheep	AB189670	Japan
HuB/2009	-	GU320351	China
Cro-Goat-11727/10	Goat	HQ215588	Croatia
Cro-Cres-12446/09	Sheep	HQ215589	Croatia
NZ2	Sheep	AY453667	New Zealand
Tillquist Pseudocowpox	Cow	AY424972	-
D1701	Reindeer	AY453654	Finland

"-": unknown

Comparisons of the obtained nucleotide sequences with those of parapoxviruses available in the Genbank database were performed using the online BLAST program. Sequence identities of nucleotides, as well as those of amino acids, were analyzed using the ClustalX implemented in Mega4 software [24]. The same tool was used to perform Neighbor-Joining (NJ) analysis, based on p-distance. Reliabilities of phylogenetic relationships were evaluated using nonparametric bootstrap analysis [25] with 1000 replicates for NJ analysis. Estimation of the Mean Evolutionary Diversity among our and vaccinal viruses were calculated as percentage of different nucleotides along a 498 nt of analysed segment using the Jukes-Cantor method in MEGA4 [26]. To confirm the obtained data from NJ analysis, another phylogenetic tree was calculated using MrBayes v3.0b3 [27]. In Bayesian Inference (BI) analysis [28] four incrementally heated Markov Chains were run for 1,000,000 generations (ngen = 1,000,000), sampling every 100 generations (samplefreq = 100), where 2500 samples were discarded (burnin = 2500). A consensus tree was constructed from the tree output files produced in the BI analysis using TreeView http://taxonomy.zoology.gla.ac.uk/rod/rod.html.

## Competing interests

The authors declare that they have no competing interests.

## Authors' contributions

IL: Study design, laboratory work and data analyses, manuscript write-up. ZC and AB: Field work, laboratory studies, manuscript preparation and proof reading. TB and BS: Field work, manuscript proof reading. ZC: Study design, manuscript proof reading and review.

All authors have read and approved the final manuscript.
